# Anti-Inflammatory, Immunomodulatory, and Tissue Repair Activity on Human Keratinocytes by Green Innovative Nanocomposites

**DOI:** 10.3390/ma10070843

**Published:** 2017-07-22

**Authors:** Pierfrancesco Morganti, Alessandra Fusco, Iole Paoletti, Brunella Perfetto, Paola Del Ciotto, Marco Palombo, Angelo Chianese, Adone Baroni, Giovanna Donnarumma

**Affiliations:** 1 R&D Nanoscience Centre MAVI, 04011 Aprilia (LT), Italy; pierfrancesco.morganti@mavicosmetics.it (P.M.); paola.delciotto@mavicosmetics.it (P.D.C.); 2 Department of Experimental Medicine, Section of Microbiology and Clinical Microbiology, University of Campania Luigi Vanvitelli, via Costantinopoli 16, 80138 Naples, Italy; alessandra.fusco@unicampania.it (A.F.); iolepaoletti@libero.it (I.P.); brunella.perfetto@unicampania.it (B.P.); 3 Plastic, Reconstructive and Aesthetic Surgery Department, CTO Hospital, 00142 Rome, Italy; palombom@virgilio.it; 4 Chemical Materials Environmental Engineering Department, University La Sapienza, 00185 Rome, Italy; angelo.chianese@uniroma1.it; 5 Multidisciplinary Department of Medical-Surgical and Dental Specialty-Dermatology, University of Campania Luigi Vanvitelli, 80138 Naples, Italy; adone.baroni@unicampania.it

**Keywords:** tissue repair, immunomodulation, green innovative nanocomposites, keratinocytes

## Abstract

The use of raw materials obtained by waste and processed through innovative industrial methodologies has generated an industry of about a trillion dollars in a short time, and in the near future will provide resources and services for the conservation and sustainable use of natural resources in order to ensure a better and fairer welfare for the human race. The production of nano-fiber chitin non-woven tissue is in accordance with the Organization for Economic Co-operation and Development (OECD) and European Union (EU) bio-economic programs: 100% biodegradable, ecological, and therefore useful in decreasing dependence on fossil fuel resources. The aim of our study is the evaluation of different formulations of a non-woven tissue obtained from electrospinning of a mixture of nanochitin fibrils, lignin, and poly (ethylene) oxide (PEO) on the restoration of damaged tissues. Wound repair is a complex process that involves epithelial and immune cells and includes the induction of metalloproteinases, inflammatory mediators, and angiogenic factors. Our in vitro results have shown that all of the realized chitin nanofibrils-bio-lignin non-woven tissues tested as nontoxic for human keratinocytes (HaCat) cells. Furthermore, the bio-composites that included bio-lignin at 0.1% have been able to modulate the expression of pro-inflammatory cytokines (Tumor Necrosis Factor-α, IL-1α, and IL8), lipopolysaccharide (LPS)-induced, and matrix metalloproteinases (MMPs) and human beta-defensin 2 (HBD-2) expression in HaCat cells, suggesting an anti-inflammatory and immunomodulatory role. Taken together, our results suggest that our chitin nanofibrils-bio-lignin non-woven tissue represents a skin-friendly tool that is able to favor a correct and fast wound repair.

## 1. Introduction

The development of bio-nanotechnology has allowed the use of marine bioproducts such as sponges, shellfish, algae, and food waste as raw materials to be transformed into value-added goods, with a low consumption of energy and water [1,2,3], producing different new green nanocomposites employed in the fields of electrochemistry, electronics, and medicine [4,5,6]. Among the natural biopolymers widely used in the biomedical field, there are chitin, chitosan, and lignin [7,8]. Chitin is a highly available natural polymer which is safe and non-toxic, and is used as nanocrystals or nanofibers [9,10]. It can be used as a versatile template for biomimetic polymers [2]. Lignin is a substance with a complex chemical structure that is easy-to-find as raw material, but is very difficult to find a purification method that allows the compound to be obtained without impurities. Lignin separation methods from natural raw materials (especially wood) involve its removal by dissolving, using suitable chemical compositions based on inorganic materials or organic solvents in the presence of catalysts and subsequent precipitation of the resulting lignin derivatives. Lignin can be used in the manufacture of biomaterials, phenolic resins, biodegradable polymer compositions, active biosorbants, surfactants and dispersant agents, and in electrochemistry. One important application of lignin is as a potential adsorbent of dangerous metal ions, such as Pb(II) > Cu(II) > Cd(II) > Zn(II) > Ni(II). In addition, attention must be paid to the two types of functional carboxylic and phenolic groups present on the lignin surface [11,12,13].

Our research group has developed and produced an advanced non-woven tissue using green nanocomposites of chitin nanofibrils (CN) electroctrospun with bio-lignin and poly (ethylene) oxide (PEO). These preparations have been tested for different applications, such as green technology membranes for water filtration or medical masks [14,15]. Here we propose a new application of the non-woven tissues produced, as a tool that could be used during wound healing to protect the tissue damage against any infections and accelerate the healing process. Wound repair often leads to incorrect scarring with the presence of tissue hypertrophy or even to non-resolution of skin damage due to bacterial infections. The scarring process requires the remodeling of the extracellular matrix (ECM), a complex protein and polysaccharide structure. In homeostasis condition and/or during tissue repair, complex changes take place in both its organization and composition [16,17]. The matrix reorganization process is guaranteed by specific enzymes, such as matrix metalloproteinases (MMPs) [18,19].

MMPs play an important role in both normal and pathological tissue repair, fibrotic repair, epithelial regeneration, and scarring remedies. During the healing process, the expression of MMPs is closely regulated by pro-inflammatory cytokines, and is altered as a result of changes in the secretion of ECM components [20].

Along with antimicrobial peptides, pro-inflammatory cytokines—particularly human beta-defensin 2 (HBD-2)—are able to regulate the expression of MMPs. In addition to the well-known antimicrobial activity, HBD-2 demonstrated an immune-modulatory effect and an important involvement in the angiogenesis and wound healing processes [21].

The purpose of our work has been to provide an in vitro demonstration of the safety, functionality, and efficacy of different non-woven chitin-bio-lignin copolymers—with a particular interest in the bio-lignin component—on the acceleration of tissue damage repair, and to obtain a better environmentally-friendly device that favors a timely wound closure that could dramatically reduce the risk of infection and keloids.

## 2. Results

### 2.1. Morphology of the CN-Bio-Lignin Nanoparticles and the Bio-Nano-Composite Non-Woven Tissues

**Figure 1:**
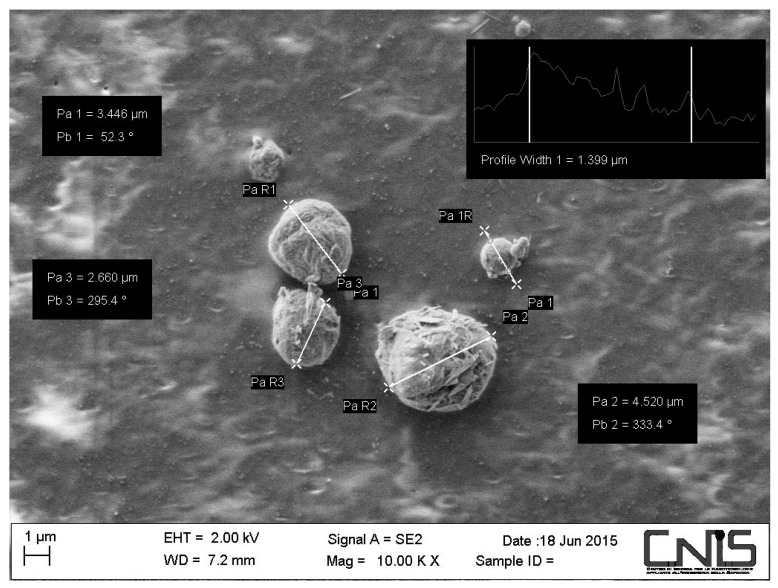
Morphology of the Chitin Nanofibril bio-Lignin micro/nanoparticles at SEM microscopy. Magnification 1000KX. From: Morganti et al. J. of Clinical and Cosmetic Dermatology Vol.1:1, 2017; Open Access [22].

**Figure 2:**
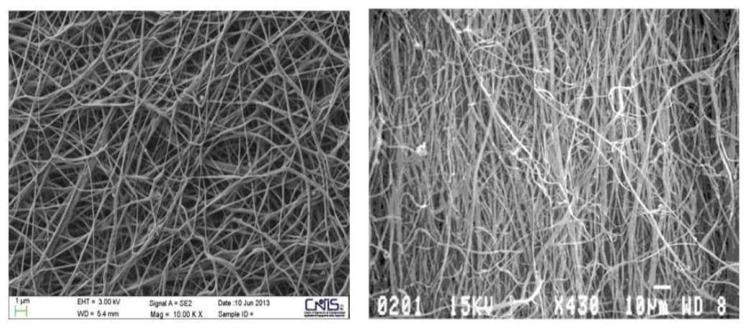
Porous structure of bio-composite Chitin Nanofibril scaffold (left) Extracellular Matrix (ECM)-like (right) at SEM microscopy. From: Morganti et al. J. of Clinical and Cosmetic Dermatology Vol.1:1, 2017; Open Access [22].

SEM observation of the chitin nanofibers-bio-lignin co-polymer showed that bio-lignin was intimately incorporated into CN-nanocrystals in the form of micro/nano balls (Figure 1). About 90% of the balls had a mean size of 61.90 nm [15], and they gave the particles’ surface a soft and regular granular appearance. In addition, nanofibers randomly assembled within the bio-nano-composite non-woven tissue gave it a morphology very similar to the human extracellular matrix (ECM) (Figure 2).

### 2.2. In Vitro Effectiveness of CN-Bio-Lignin Non-Woven Tissues on Cell Viability

In vitro studies demonstrated that the particular morphology and composition of the CN-bio lignin non-woven tissue gives it a great cell-affinity. Here we evaluated the effects of this advanced medication on human keratinocytes. MTT assay demonstrated that all the copolymers tested at different concentrations of 100, 50, and 10 µg/mL did not affect HaCat cells’ viability (Table 1).

### 2.3. In Vitro Effectiveness of CN-Bio-Lignin Non-Woven Tissues on HBD-2 and MMPs Release from HaCat Cells

**Figure 3:**
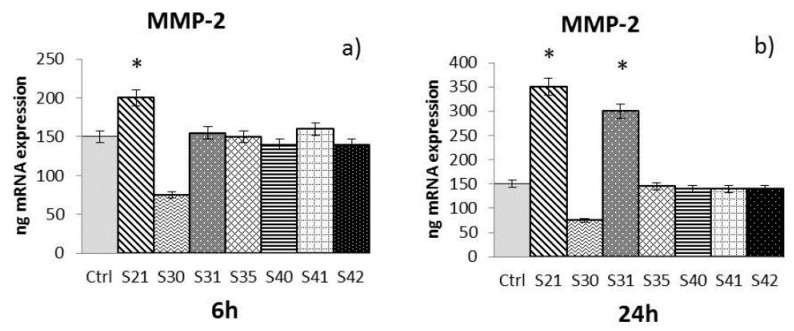
mRNA expression of Metalloproteinase-2 (MMP-2) from HaCat cells treated with S21, S30, S31, S35, S40, S41 and S42 non-woven tissue bio-composites (10 μg/mL) for 6 h (**a**) and 24 h (**b**). Data are mean ± SD of values calculated on two different experiments. * *P* < 0.05 compared to Ctrl.

**Figure 4:**
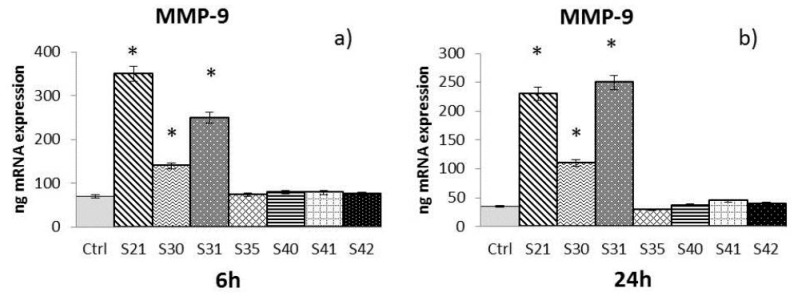
mRNA expression of Metalloproteinase-9 (MMP-9) from HaCat cells treated with S21, S30, S31, S35, S40, S41 and S42 non-woven tissue bio-composites (10 μg/mL) for 6 h (**a**) and 24 h (**b**). Data are mean ± SD of values calculated on two different experiments. * *P* < 0.05 compared to Ctrl.

**Figure 5:**
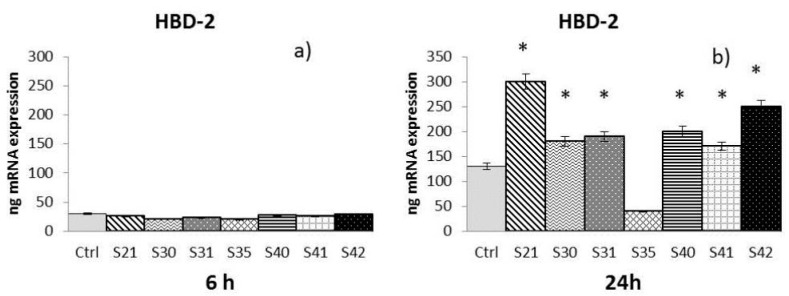
mRNA expression of HBD-2 from HaCat cells treated with S21, S30 , S31, S35, S40, S41 and S42 non-woven tissue bio-composites (10 μg/mL) for 6 h (**a**) and 24 h (**b**). Data are mean ± SD of values calculated on two different experiments. * *P* < 0.05 compared to Ctrl.

The samples S21, S30, S31, S35, S40, S41 and S42 of non-woven tissue at a concentration of 10 µg/mL—chosen as the best concentration in preliminary experiments (data not shown)—were tested for their ability to induce MMP-2, MMP-9, and HBD-2 from HaCat cells after 6 h and 24 h of treatment:

- MMP-2: At 6 h (Figure 3a), only S21 gave a significant enhancement of MMP-2, while at 24 h (Figure 3b) both S21 and S31 showed an increase of MMP-2 expression compared to control.
- MMP-9: After both 6 h and 24 h (Figure 4a,b), only S21, S30 and S31 among the bio-composites tested showed a significant increase of MMP-9 compared to control.
- HBD-2: At 24 h (Figure 5b), all the samples tested with the exception of S35 showed a significant enhancement of HBD-2 expression compared to control. No significant results were obtained for HBD-2 at 6 h (Figure 5a).

The Real-Time Polymerase Chain Reaction (RT-PCR) results have been expressed as the mean value ± SD of mRNA expressions of the gene of interest, resulting from two independent experiments in triplicate.

### 2.4. In Vitro Effectiveness of CN-Bio-Lignin Non-Woven Tissues on Inflammatory Cytokines Released from HaCat Cells Treated with Lipopolysaccharide (LPS)

**Figure 6:**
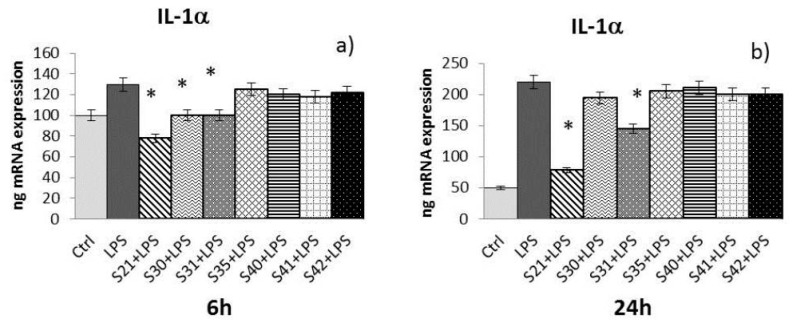
mRNA expression of IL-1α from HaCat cells treated with S21, S30 , S31, S35, S40, S41 and S42 non-woven tissue bio-composites (10 μg/mL) plus *P. aeruginosa* lipopolysaccharide (LPS) (10 μg/mL) 6 h (**a**) and 24 h (**b**). Data are mean ± SD of values calculated on two different experiments. * *P* < 0.05 compared to LPS (10 μg/mL) alone.

**Figure 7:**
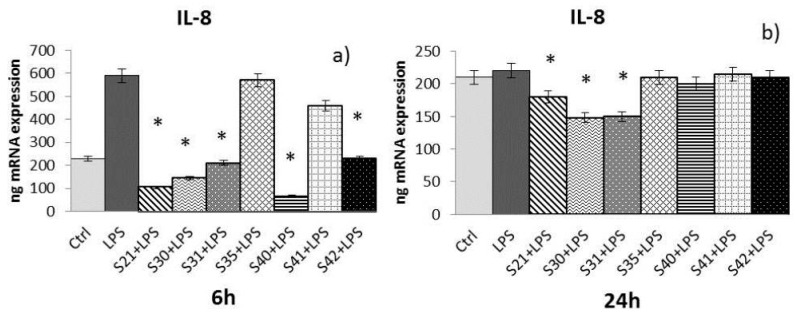
mRNA expression of IL-8 from HaCat cells treated with S21, S30 , S31, S35, S40, S41 and S42 non-woven tissue bio-composites (10 μg/mL) plus *P. aeruginosa* lipopolysaccharide (LPS) (10 μg/mL) 6 h (**a**) and 24 h (**b**). Data are mean ± SD of values calculated on two different experiments. * *P* < 0.05 compared to LPS (10 μg/mL) alone.

**Figure 8:**
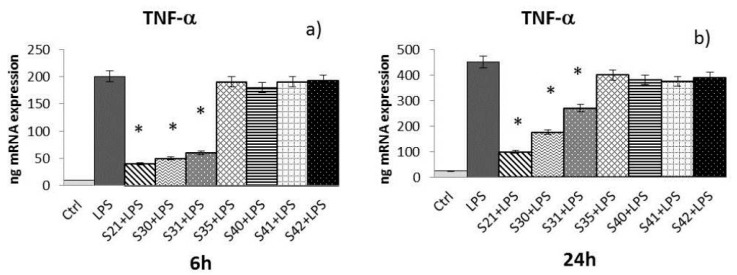
mRNA expression of TNF-α from HaCat cells treated with S21, S30 , S31, S35, S40, S41 and S42 non-woven tissue bio-composites (10 μg/mL) plus *P. aeruginosa* lipopolysaccharide (LPS) (10 μg/mL) 6 h (**a**) and 24 h (**b**). Data are mean ± SD of values calculated on two different experiments. * *P* < 0.05 compared to LPS (10 μg/mL) alone.

Samples S21, S30, S31, S35, S40, S41 and S42 of non-woven tissue at a concentration of 10 µg/mL (chosen as the best concentration in preliminary experiments, data not shown) were tested for their ability to reduce cytokine expression (e.g., IL-1α, IL-8, and TNF-α) induced by LPS (10 µg/mL) in HaCat cells after 6 h and 24 h of treatment:

- IL-1α: At 6 h (Figure 6a) of treatment, only S21, S30, and S31 were able to reduce IL-1α induced by LPS, while at 24 h (Figure 6b), only S21 and S31 maintained the effect.
- IL-8: At 6 h (Figure 7a) of treatment, S21, S30, S31, S40, and S42 showed a reduction of LPS-induced IL-8. After 24 h of treatment (Figure 7b), only S21, S30, and S31 gave a significant reduction of IL-8 induced by LPS.
- TNF-α: After both 6 and 24 h (Figure 8a,b) of treatment, only S21, S30, and S31 showed a significant reduction of TNF-α induced by LPS.

The Real-Time Polimerase Chain Reaction (RT-PCR) results have been expressed as the mean value ± SD of mRNA expressions of the gene of interest, resulting from two independent experiments in triplicate.

### 2.5. Statistical Analysis

Each experiment was performed at least two times in triplicate. The data obtained have been analyzed using Student’s t-test. A *P*-value < 0.05 has been considered statistically significant.

## 3. Discussion

Natural nano-fibers of chitin and bio-lignin are frequently used to develop multifunctional materials for different uses (e.g., drug delivery devices). Such devices are easily biodegradable, thus avoiding their removal after application in biological systems [4,7,14]. Chitin is a polymer made of glucosamine and acetyl-glucosamine, and it is important to emphasize that because it is a hyaluronic acid, it possesses the ability to bind large amounts of water (a key element for all cellular activities), and that the human chitotriotidases are easily metabolized [22,23].

Bio-lignin is also an interesting molecule with antioxidant properties that consists of monomers of polyphenol [24,25]. Lignins are highly-branched phenolic molecules of vegetal origin, and have phenolic groups possessing hydroxyl, carboxyl, carbonyl, and methoxyl groups which confer important properties, such as antioxidant and antibacterial activity. In addition, CN-bio-lignin—and in particular the described electrospun tissues—are able to carry cargo such as nanosilver and other selective active ingredients which are necessary to prevent infection. In addition, it is easily resorbable because of its hierarchical structure identical to the macromolecules present in the ECM, and is easily electrospun by an economical method [15]. Garcia et al. emphasized the importance of the fractionation process on the lignin purity and structure to obtain lignins with different efficiency [26].

Wound matrix plays a key role as the regulator of cell adhesion, migration, proliferation, and differentiation during repair. Our attention has been focused on the role of the gelatinase A and B, MMP-2, and MMP-9, respectively, during the wound repair process. These type IV collagenases are secreted by keratinocytes, and have catalytic activity binding to gelatin, collagens, and laminin. The gelatinases, MMP-2 and MMP-9, are involved in wound repair; the former during ECM remodeling and the latter in epithelialization. Different kinds of cells releasing gelatinases in the wound environment are therefore consistent with their role in wound healing. Wound repair is a complex process consisting of different phases: MMP-9 seems to be involved in the first phase (the inflammatory phase), while an increased level of MMP-2 is evident during the development of the granulation phase and remains high during the entire phase of ECM remodeling. In addition, both play an important role during the processes of micro-vessel maturation and in scar tissue remodeling, to reestablish the normal and functional architecture of the damaged tissue. It is well known that gelatinases also act as modulators of inflammation [20,27].

Inflammation activates the expression of a gene product cascade that results in the release of several pro-inflammatory cytokines, such as IL-8, TNF-α, IL-1, and others. IL-8 induces phagocytosis [28], causes tissue damage, and is involved in the degranulation of neutrophils, while TNF-α induces cachexia, fever, triggers cells in apoptosis, and inhibits viral replication [29]. During infectious processes, trauma, or ischemia, IL-1 and TNF-α production synergistically lead to the cascaded activation of inflammatory mediators. However, pro-inflammatory cytokines are also involved in regulating the expression of antimicrobial peptides. These are constitutive or inducible products of innate immunity which are able to act broadly against viruses, bacteria, and fungi; one example is HBD-2. HBD-2 is an inducible peptide with antimicrobial activity which is present in psoriatic lesions, but also in other epithelia [30,31]. It is able to stimulate angiogenesis [32], histamine release from mast cells, and induces synthesis of prostaglandin. In addition, HBD-2 shows pro-inflammatory properties because it is a chemo-attractant for memory T-cells, neutrophils, mast cells, and immature dendritic cells.

We demonstrated via in vitro experiments that all the realized CN-bio-lignin non-woven tissues tested non-toxic for HaCat cells. In addition, the bio-composite S35—without the presence of bio-lignin—was unable to modulate MMPs, HBD-2, and the inflammatory cytokines evaluated. Among the bio-composites tested, those with a concentration of lignin at 0.1% (S21, S30, and S31)—particularly S21—were able to modulate MMPs, HBD-2, and LPS-induced pro-inflammatory cytokines in HaCat cells. These data suggest that non-woven tissue, particularly those that contain lignin at 0.1%, can favor the mechanisms of remodeling and accelerate tissue repair by operating on the modulation of MMPs, reinforcing the cyto-protective response under stress conditions. In addition, collagenase stimulation could also reduce fibrous tissue production, suggesting that non-woven tissue is effective under conditions with an overproduction of collagen, such as keloid scars. From histologic analysis, keloids appear as a hyper-proliferation of the fibroblasts from reticular and sometimes papillary dermis, with consequent densely-packed collagen fibers and an absence of elastic tissue. The fiber welds into hyaline cords and fibrous connective tissue fills the gaps between the meshwork [33]. Thus, CN-bio-lignin non-woven tissue may also be a helpful device for the prevention and treatment of keloids. Their ability to reduce the expression of LPS-induced pro-inflammatory cytokines such as IL-1α, IL-8, and TNF-α and increase expression of HBD-2 in human keratinocytes proves that the realized CN-bio-lignin non-woven tissues show significant anti-inflammatory and immunomodulatory properties. These pharmacological properties are accompanied by an increased mechanical resistance of the CN bio-composites due to the presence of lignin [15].

Thanks to its composition of natural recycled raw materials, our nonwoven tissue is 100% biodegradable and is therefore compatible with the environment and in accordance with the OECD and EU programs and bio-economy. CN-bio-lignin non-woven tissue is a valuable resource that decreases dependence on fossil fuel resources and is a valid medical tool which is skin-friendly and able to favor a correct and fast wound repair.

## 4. Materials and Methods

### 4.1. Materials and Non-Woven Tissue Preparation

MAVI SUD Srl (Aprilia, Latina, Italy) provided the chitin nanofibrils in a 2% water suspension and Ag nanostructures, CIMV (Neuilly sur Seine, France) for bio-lignin. PEO was purchased from Amerchol Dow Italia, (Milan, Italy), polypeptides from Roussel Sas (Pateaux, France), and chitosan from Giusto Faravelli Spa (Milan, Italy). For non-woven tissue preparation, briefly: CN-bio-lignin was aggregated in polymeric nanoparticles containing lignins obtained with different purification methods and reported as Lignin, Lignin I, and Lignin II, using a DF 500 B9 spray-dryer (JCF, Milano, Italy) and dissolved into deionized water at 15 °C. PEO was added to the obtained gel-mixture used as starting material for electrospinning on a bed of polypropylene; the electrospinning process was performed by using an Elmarco Nanospider NS LAB 500 pilot-scale machine based on the nozzleless technology, as previously reported [14,15,34].

### 4.2. Characterization of Nanoparticles and Non-Woven Tissues

A field emission electron microscope-FESEM Auriga Zeiss (Carl Zeiss, Milano, Italy) with Energy Dispersive X-Ray Spectrometry microanalysis EDS 123 Mn-Ka eV (Bruker) and Electron Bean Lithography EBL–7 nm resolution (Raith) was used to characterize the surface morphology of electrospun nanofibers. Samples from the electrospun material were mounted on aluminum stubs and coated with an ultrathin layer of platinum to obtain a better conductivity during the microscopic observation. Magnifications between 100 and 40,000 times the original sizes were used to visually evaluate the electrospinnability and the existence of beads. ImageJ image processing software was used to evaluate fiber diameters; three different images for each electrospun material (at least 100 fibers) were analyzed to calculate the average diameter.

### 4.3. Cell Culture

Human keratinocyte HaCat cells (from the American Type Culture Collection, ATCC) cultured in Dulbecco’s Modified Eagle’s Medium (DMEM), supplemented with 10% (*v*/*v*) heat-inactivated fetal calf serum (FCS), a standard mixture of 100 U mL^−1^ penicillin/100 µg mL^−1^ streptomycin, and 1% l-glutamine were maintained at 37 °C with 5% CO_2_. All reagents were purchased from Gibco-BRL (Thermo Fisher Scientific, Waltham, MA, USA, 02451). HaCat cells were cultured in six-well plates (Becton Dickinson GmbH, Heidelberg, Germany) at a concentration of 1 × 10^6^ cells/well. Before treatments, semi-confluent monolayers were starved with serum-free DMEM in order to stop the growth and make a synchronous cell culture.

### 4.4. Cell Treatments

Different types of biocomposite non-woven tissues made of chitin nanofibrils-Ag/bio-lignin, chitosan/polypeptides/PEO were provided by MAVI SUD. The bio-composites differ in the type and concentration of bio-lignin, as reported in Table 2.

For the experiments, the hydro-soluble non-woven tissue was solubilized in phosphate-buffered saline (PBS), obtaining a solution with a final concentration of 100 µg/mL. Samples that showed a pH of 9.0 upon solubilization were also brought to physiological pH (7.0) and sterilized by autoclaving.

For the experiments, the human keratinocytes were treated with the samples used at final concentrations of 100, 50, and 10 µg/mL. For the evaluation of the inflammatory activity, cells were stimulated with samples in the presence or absence of 10 µg/mL of *Pseudomonas aeruginosa* LPS. Treatments were carried out in triplicate for 6 and 24 h, and two independent experiments were performed.

### 4.5. MTT Proliferation Assay

HaCat cells (3 × 10^3^ cells/well) seeded in microplates (96 wells, flat bottom) in a final volume of 100 µL DMEM per well were treated with the samples at 100, 50, and 10 µg/mL, and incubated at 37 °C, 5% CO_2_ for 72 h. After the incubation, 10 µL of MTT labelling reagent (Sigma-Aldrich, Milan, Italy) at a final concentration of 0.5 mg/mL was added to each well for 4 h. Then, 100 µL of the solubilized was added to each well and plates were incubated overnight. Spectrophotometric absorbance was measured using a microplate ELISA reader (Biorad, Milan, Italy) at a wavelength of 570 nm.

### 4.6. Real-Time PCR

HaCat 1 × 10^6^ cells/well seeded in microplates (six wells) in a final volume of 1 mL DMEM per well were treated with the samples at the following concentrations: 100, 50, and 10 µg/mL. For evaluation of cytokines, the treatments were performed in the presence or absence of 10 µg/mL of *P. aeruginosa* LPS. Cells were incubated at 37 °C, 5% CO_2_ for 6 h and 24 h. A high-purity RNA isolation kit (Roche; Milano, Italy) was used to extract total RNA from HaCat cells. One microgram of it was reverse-transcribed at 37 °C for 1 h into complementary DNA (cDNA) using M-MLV reverse transcriptase (Promega; Milano, Italy) and random hexamer primers (random hexamers, Roche; Milano, Italy), according to the manufacturer’s instructions. RT-PCR for HBD-2, MMP-9, MMP-2, IL-8, IL-1α, and TNF-α were performed with an LC Fast Start DNA Master SYBR Green kit (Roche; Milano, Italy) using 2 µL of cDNA (100 ng of total RNA) in a 20 µL final volume containing 3 mM MgCl_2_ and 0.5 mM sense and antisense primers (Table 3). At the end of each amplification, a melting curve was made to ensure the absence of non-specific reaction products. The accuracy of mRNA quantification depends on the linearity and efficiency of the PCR amplification. Both parameters were assessed using standard curves generated by increasing the amounts of cDNA. Quantification is based on the threshold cycle values, which are measured in the early stage of the exponential phase of the reaction.

## 5. Conclusions

The nanofibers of CN and bio-lignin, made by natural raw materials obtained from biomass, find application as non-woven tissues, that has shown to be skin friendly and 100% biodegradable reducing the dependence from fossil fuel resources. The obtained results by the formulations of non-woven tissue tested are very encouraging for the application of these materials as biomedical tool able to favor the mechanisms for accelerating damaged tissue repair, by acting on the modulation of MMPs, cytokines and antimicrobial peptide HBD-2 thus reinforcing the cyto-protective response under stress conditions.

## Figures and Tables

**Table 1:** Optical density (O.D.) reading at 570 nm resulting from the MTT assay.

Sample	Concentration (10 µg/mL)	O.D.
Ctr		1.56
S35	10	1.7
	50	1.5
	100	1.7
S21	10	1.4
	50	1.3
	100	1.35
S40	10	1.6
	50	1.56
	100	1.61
S42	10	1.35
	50	1.36
	100	1.38
S41	10	1.71
	50	1.69
	100	1.5
S30	10	1.3
	50	1.2
	100	1.26
S31	10	1.3
	50	1.36
	100	1.4

**Table 2:** Chitin nanofibrils-Ag/bio-lignin, chitosan/polypeptides/poly (ethylene) oxide (PEO) non-woven tissues.

Samples	Main Composites		
S35	PEO 7%	CN 30% (*w*/*w*)	/////
S21	PEO 7%	CN 30% (*w*/*w*)	Lignin 0.1%
S40	PEO 7%	CN 30% (*w*/*w*)	Lignin 1%
S42	PEO 7%	CN 30% (*w*/*w*)	Lignin 2.5%
S41	PEO 7%	CN 30% (*w*/*w*)	Lignin 5%
S30	PEO 7%	CN 30% (*w*/*w*)	Lignin I 0.1%
S31	PEO 7%	CN 30% (*w*/*w*)	Lignin II 0.1%

**Table 3:** Primer sequences and amplification conditions.

Gene	Primers Sequence	Conditions	Product Size (bp)
IL-8	5-ATGACTTCCAAGCTGGCCGTG-3′	5″ at 94 °C, 6″ at 55 °C,	297
	5-TGAATTCTCAGCCCTCTTCAAAAACTTCTC-3	12″ at 72 °C for 40 cycles	
IL-1 α	5′-CATGTCAAATTTCACTGCTTCATCC-3′	5″ at 95 °C, 8″ at 55 °C,	421
	5′-GTCTCTGAATCAGAAATCCTTCTATC-3′	17″ at 72 °C for 45 cycles	
TNF- α	5′-CAGAGGGAAGAGTTCCCCAG-3′	5″ at 95 °C, 6″ at 57 °C,	324
	5′-CCTTGGTCTGGTAGGAGACG-3′	13″ at 72 °C for 40 cycles	
MMP-2	5′-TGACGGTAAGGACGGACTC-3′	5″ at 94 °C, 7″ at 57 °C,	342
	5′-TGGAAGCGGATTGGAAACT-3′	14″ at 72 °C for 40 cycles	
MMP-9	5′-GCCAACTACGACACCGACGA-3′	5″ at 95 °C, 6″ at 56 °C,	310
	5′-CGCTGGTACAGGTCGAGTAC-3′	12″ at 72 °C for 40 cycles	
HBD-2	5′-GGATCCATGGGTATAGGCGATCCTGTTA-3′	5″ at 94 °C, 6″ at 63 °C,	198
	5′-AAGCTTCTCTGATGAGGGAGCCCTTTCT-3′	10″ at 72 °C for 50 cycles	
